# Functional connectivity profiles of amygdala subregions in posttraumatic stress disorder

**DOI:** 10.1038/s41398-025-03508-y

**Published:** 2025-08-14

**Authors:** Elizabeth M. Haris, Richard A. Bryant, Kim L. Felmingham, Leanne M. Williams, Mayuresh S. Korgaonkar

**Affiliations:** 1 Brain Dynamics Centre, Westmead Institute for Medical Research, The University of Sydney, Westmead, NSW Australia; 2 School of Psychology, University of New South Wales, Sydney, NSW Australia; 3 School of Psychological Sciences, Department of Medicine, Dentistry and Health Sciences, The University of Melbourne, Parkville, VIC Australia; 4 Department of Psychiatry and Behavioral Sciences, Stanford University School of Medicine, Stanford, CA USA; 5 Mental Illness Research, Education and Clinical Center, VA Palo Alto Health Care System, Palo Alto, CA USA; 6 Discipline of Psychiatry, Sydney Medical School, Westmead, NSW Australia; 7 Department of Radiology, Western Sydney Local Health District, Westmead, NSW Australia

**Keywords:** Human behaviour, Diagnostic markers

## Abstract

The amygdala is crucial to understanding posttraumatic stress disorder (PTSD), yet knowledge of the connectivity of its substructures and their contribution to the functional heterogeneity characteristic of the disorder remains limited. This study sought to delineate the functional profiles of amygdala substructures to advance a more nuanced understanding of their contribution to the neural circuitry underlying PTSD in a large sample. Task-derived intrinsic functional magnetic resonance imaging (rs-fMRI) data for 64 non-trauma-exposed controls (NEC) and 65 individuals with PTSD were analyzed. Amygdala subnuclei were segmented using FreeSurfer and combined into three major substructures for each hemisphere: the basolateral (BLA), centromedial (CMA), and superficial (SFA) nuclei. Seed-to-voxel functional connectivity analyses for the whole brain were performed to investigate group differences in subnuclei connectivity profiles. A significant group by subnuclei interaction was found for four clusters, driven by group differences in connectivity related to the BLA. There was *lower* connectivity in the PTSD group for the left BLA and precuneus, posterior cingulate, right superior parietal lobe, right postcentral gyrus, and bilateral precentral gyri. *Higher* connectivity was found in the PTSD group for the left BLA and brainstem, and for the right BLA and cerebellum, and brainstem and right pallidum. No group differences were found for the CMA or SFA. These results illustrate the importance of the BLA in driving task-derived intrinsic functional connectivity between NEC and PTSD groups. Findings suggest that group differences lie in *lower* connectivity with cortical areas involved in self-referential and sensorimotor processing, but *higher* connectivity with subcortical areas involved in arousal, salience, sensory, and reward processing.

## Introduction

The amygdala is paramount in our understanding of the neural circuitry underlying emotional processing [1] and psychopathology, and known for its crucial role in fear memory and responses [2, 3]. It is consistently implicated in the neurobiology of posttraumatic stress disorder (PTSD; [4, 5]), a disorder characterized by fear processing impairments. Meta-analyses investigating functional connectivity in PTSD have found hyperconnectivity between the amygdala and insula—regions involved in detecting stimuli salience [6], and decreased connectivity in the default mode network (DMN)—interconnected regions involved in self-referential processing at rest [4, 6].

Located bilaterally in the anteromedial temporal lobes, the amygdala can be divided into nine nuclei in humans [7], which are often grouped together into three larger structures based on similar cytoarchitecture and functionality [8, 9]. The basolateral amygdala (BLA) includes the deeper subnuclei and is the largest of the three subnuclei. Rodent and non-human primate work shows reciprocal connections between the BLA and medial/orbital prefrontal areas, memory and reward processing regions, and sensory association areas [10, 11]. It is involved in associative learning, particularly the acquisition of conditioned fear [12], integration of somatosensory and affective information [13], and promotion of reward-seeking behaviors [14]. The centromedial amygdala (CMA), the smallest of the three nuclei, receives most of its afferent projections from the rest of the amygdala [11] and primarily projects to brainstem regions [15]. It is involved in the facilitation of behavioral responses, particularly fear extinction [12], essentially converting sensory information into behavioral change [11]. Less is known about the connectivity of the superficial amygdala (SFA), however, it has been shown to be connected to the olfactory cortex and to have a functional role in olfaction, audition, affective, and social processing [11, 16, 17].

Most PTSD studies treat the amygdala as a single region [4, 5, 18], potentially masking findings by averaging signals across its functionally distinct subnuclei. Only 11 studies have investigated amygdala subnuclei functional connectivity, with just nine examining intrinsic functional connectivity using resting-state functional magnetic resonance imaging (for a review, see, [9]). Of these, only two reported overlapping results. Greater connectivity in PTSD was observed between the right BLA and right dorsal anterior cingulate relative to trauma-exposed controls (TEC); and between the left BLA and right middle frontal gyri relative to non-trauma-exposed controls (NEC). While this highlights the importance of the comparison group in PTSD research, it also highlights the need for more research investigating amygdala subnuclei connectivity patterns in PTSD in general to understand their contribution to the neural circuitry underlying the disorder.

Resting-state fMRI studies show that BLA, CMA, and SFA intrinsic functional connectivity patterns involve regions that are part of the cognitive control, default mode, and salience networks—all networks that show disruption in PTSD [4, 6]. Yet, two key limitations persist: (1) except for three studies [19–21], all others comprised fewer than 30 PTSD participants, potentially limiting reliability, and (2) previous studies used a variety of standardized atlases (which provide normalized amygdala subnuclei templates) rather than using subject-specific templates [7]. While subject-specific templates are increasingly employed to investigate subcortical structure in PTSD [22, 23], they have yet to be applied to investigate amygdala subnuclei functional connectivity in PTSD.

To address prior limitations, we analyzed previously obtained task-derived intrinsic functional connectivity data of the BLA, CMA, and SFA in a larger PTSD sample alongside a comparatively sized NEC group. Although not ‘pure’, task-free resting-state data, task-derived intrinsic functional connectivity closely mirrors resting-state networks and offers insight into the interaction of regions with these networks [24–26]. To better understand the neural circuitry underlying PTSD, we also employed subject-specific amygdala subnuclei delineation to measure activity and connectivity of each amygdala subregion—a novel approach in this context. Based on prior literature, we expected to find differences group differences in subnuclei functional connectivity with areas involved in attention, reward, sensory, and self-referential processing networks. Specifically, we anticipated greater connectivity in PTSD between the BLA and middle and medial frontal areas (dorsal anterior cingulate cortex), and possibly between the CMA and superior frontal/temporal areas and middle occipital gyri, and between the SFA and fusiform, lingual, and middle occipital gyri. We also hypothesized reduced connectivity in PTSD between the BLA and superior temporal and frontal regions, and possibly between the CMA and middle and medial frontal areas (ventromedial prefrontal cortex), and the thalamus.

## Materials and methods

### Participants

MRI data for 68 non-trauma-exposed controls (NEC) and 67 participants with PTSD were initially analyzed. A diagnosis of PTSD was determined using the Clinical Administered PTSD Scale (CAPS; [27]), a structured clinical interview based on criteria from the Diagnostic and Statistical Manual of Mental Disorders (4th ed) [28]. Clinical participants were civilians who had experienced various mixed trauma, with 48% experiencing childhood trauma. Comorbidities included major depression (MDD; 49%), generalized anxiety (GAD; 28%), and eating disorders (14%). Inclusion criteria comprised those aged at least 18 years and medication free or on a stable dose of psychotropic medication for the past two months. Participants with PTSD were excluded if they had a history of neurological disorder, traumatic brain injury, psychosis, or substance dependence. Four NEC and two PTSD participants were excluded at analysis stage due to technical issues with amygdala subnuclei segmentation and normalization, leaving a total of 64 NEC and 65 PTSD participants to be analyzed.

The study complied with the Declaration of Helsinki 1975, as revised in 2008. Procedures were approved by the Western Sydney Area Health Service Human Ethics Committee. Written informed consent was collected from all participants.

### Image acquisition and preprocessing

This study analyzed task-derived intrinsic connectivity data. Details regarding the acquisition and pre-processing of fMRI data have been previously described [29]. Functional MRI data was acquired on a 3T GE Signa scanner with a phased array eight-channel head coil. Structural T1-weighted SPGR images and data from five EPI fMRI tasks were obtained. Cognitive tasks used an event-related design (task 1), while emotion-related tasks used a mixed block/event-related design (tasks 2–4). Tasks included: (1) a Go-NoGo task measuring impulsivity/inhibition, where participants were to respond to the green word PRESS, displayed for 500 ms; (2) two runs of a cognitive appraisal task measuring emotion reappraisal (first run) and emotional reactivity (second run), where participants either passively viewed or were required to down-regulate their emotional response to traumatic or neutral images (three blocks (Think, Neutral, Watch) of 10 images, each shown for 10 s) and to rate how negative these images made them feel; and (3/4) an unmasked conscious emotion processing task measuring explicit/conscious processing of emotions, and a masked non-conscious emotion processing task measuring implicit/unconscious processing of emotions, where both tasks involved the presentation of six faces with different emotional expressions—eight different faces with the same emotion were presented for 500 ms in a block design and repeated five times (backward masking of 10 ms followed by a neutral expression of 490 ms was used for the unconscious task; all tasks described in detail in, [30]). Data were pre-processed using SPM8 in MATLAB [31]. Images were realigned and unwarped, and quality control comprised the removal of average white matter and cerebrospinal fluid signal, and identification and removal of movement outliers (realignment parameters with Volterra expansion [32]; framewise displacement ≥0.3 mm, and slice-to-slice signal intensity differences >10 (using the SPM8 TSDiffAna toolbox); two volumes before/one volume after these two latter parameters were also scrubbed [33]). A temporal mask was created for each censored and subsequent volume and used as a nuisance regressor in first-level statistical models. Motion-corrected images were slice-time corrected, spatially normalized to 2 mm MNI space, and smoothed using an 8 mm FWHM Gaussian kernel. Task-related data was extracted by using a general linear model (GLM) to model the blood oxygen level dependent (BOLD) response for the experimental condition for all tasks: Go-NoGo task (Go and NoGo trials), cognitive reappraisal task (trial type – Think vs Watch for the first reappraisal run, Watch vs Neutral for the second emotional reactivity run), and emotion tasks (emotion type). These effects were included as covariates of no interest and removed from the task data. Intrinsic functional connectivity was estimated as the residual images from each task-related GLM and comprised 600 corrected functional volumes (120 volumes × five tasks (task two included twice given they measured different contrasts)) which were then bandpass filtered (0.009 Hz < f < 0.08 Hz). This method has been previously validated and has been found to demonstrate similar patterns of activity to those found in pure resting-state studies [34]. Of note, 12 participants (4 NEC, 8 PTSD) did not complete all tasks so had less than 600 volumes for their derived intrinsic functional connectivity images—either 360 (three tasks completed) or 480 (four tasks completed). Excluding these participants did not alter our findings (except one finding which is outlined in the results section). Therefore, data from all participants is reported.

Automated volumetric segmentation of amygdala subnuclei was performed using FreeSurfer (version 7.1.0; http://surfer.nmr.mgh.harvard.edu/; [7, 35]). Nuclei volumes were checked for major deviations, and outliers (±1.5 IQR) were visually inspected for segmentation failures. Gray matter volumes for 18 amygdala subnuclei regions of interest were extracted (nine per hemisphere): accessory-basal, anterior-amygdaloid-area, basal, central, cortical, cortico-amygdaloid-transition, lateral, medial, and paralaminar nuclei. Due to the small size of several nuclei and limited spatial resolution at 3T [36], nuclei were combined into three traditionally-defined bilateral subfields [8]: BLA (accessory-basal, basal, lateral, paralaminar nuclei), CMA (central and medial nuclei), and SFA (anterior-amygdaloid-area, cortico-amygdaloid-transition, cortical nuclei). Amygdala segments were warped from FreeSurfer to MNI space using FLIRT and FNIRT commands in FSL [37–40] to transform them into functional space. This was done using the *hires2standard* image generated by FSL during initial preprocessing to which the functional image was co-registered. Individual subnuclei masks were binarized then combined using *fslmaths* to create whole bilateral amygdalae to identify overlapping voxels. These voxels were then removed from the respective masks to create non-overlapping subnuclei masks that covered separate areas of the amygdala at an individual participant level for more accurate connectivity analyses.

### Statistical analysis

Connectivity analyses were conducted in MATLAB toolbox CONN [41, 42]. Preprocessed and task-derived intrinsic functional time series data was bandpass frequency filtered between 0.009 and 0.08 Hz. First-level, seed-to-voxel analyses generated seed-based connectivity maps for three amygdala subnuclei (separately for each hemisphere) to characterize the spatial pattern of functional connectivity with a seed area for each individual participant. Functional connectivity strength was represented by Fisher-transformed bivariate correlation coefficients from a weighted GLM [43], estimated separately for each seed area and target voxel, modeling the association between their BOLD signal timeseries.

Two group-level analyses comparing subnuclei connectivity in the left and right hemisphere, were performed using a GLM. For each individual voxel a separate GLM was estimated, with first-level connectivity measures (i.e., correlation coefficients) at this voxel as dependent variables, group and subnuclei (first analysis: left BLA, CMA, SFA; second analysis: right BLA, CMA, SFA) as independent variables, a group × subnuclei interaction, and age included as a covariate (given groups were not age-matched; Table 1). Voxel-level analyses were evaluated using multivariate parametric statistics with random-effects across subjects and sample covariance estimation across multiple measurements, thresholded at an initial voxel level of *p* < 0.001. Inferences were performed at the level of individual clusters (groups of contiguous voxels). Further cluster-level inferences were conducted based on nonparametric statistics using Threshold Free Cluster Enhancement (TFCE; [43, 44]) with 1000 residual-randomization iterations, and thresholded using a family-wise error (FWE) corrected *p*-FDR < 0.05 TFCE-score threshold.

**Table 1:** Sample characteristics and summary statistics.

	NEC (64)	PTSD (65)	Stats (W/H/*χ*^2^)	*p* value
**Age (years; Mean (SD), Median (IQR))**	35.6 (11.9) 32.8 (11.3)	39.5 (11.2) 40.5 (17.2)	1603	0.03^*^ (NEC < PTSD)
**Sex (F; %)**	46 (72)	44 (68)	0.27	0.60
**Education (No/%)**			0.50	0.48
Secondary/High School	4 (6.25)	18 (26.7)	-	-
Trade qualification	0 (0.00)	2 (3.08)	-	-
Certificate/Diploma	17 (26.6)	23 (35.4)	-	-
Graduate degree	23 (36.0)	13 (20.0)	-	-
Postgraduate degree	16 (25.0)	4 (6.15)	-	-
Other/Prefer not to answer	1 (1.56)	0 (0.00)	-	-
Not answered	3 (4.69)	5 (7.69)	-	-
**Left Hemisphere Subnuclei (mean(sd)/median(IQR) mm**^**3**^**)**	BLA > SFA > CMA	BLA > SFA > CMA	-	<0.001^**^ (wg)
Basolateral Nucleus (BLA)	2256 (320)	2248 (216)	2315	0.27
Superficial Nucleus (SFA)	384 (64)	392 (72)	2110	0.89
Centromedial Nucleus (CMA)	75.0 (24.3)	89.6 (32.6)	−2.88	0.005^*a^
**Right Hemisphere Subnuclei (mean(sd)/median(IQR) mm**^**3**^**)**	BLA > SFA > CMA	BLA > SFA > CMA	-	<0.001^**^ (wg)
Basolateral Nucleus (BLA)	2360 (328)	2384 (248)	2139	0.78
Superficial Nucleus (SFA)	405 (59.0)	402 (64.4)	0.36	0.72
Centromedial Nucleus (CMA)	93.5 (31.5)	95.9 (26.4)	−0.47	0.64

Statistical tests performed were t-tests, chi-squared test, and nonparametric Mann-Whitney U (W) and Kruskal-Wallis (H) tests, all performed in R.

*NEC* non-trauma-exposed controls, *PTSD* posttraumatic stress disorder, *F* female, *wg* within-group comparison.

^*^Significant at *p* < 0.05.

^**^Significant at p < 0.001. Within-group comparisons between three nuclei for each hemisphere were significantly different from one another.

^a^Significant after family-wise error correction for 3 comparisons per hemisphere, *p* < 0.02.

Functional connectivity results were extracted from CONN [41, 43] and separate post-hoc pairwise comparisons for significant clusters were conducted in R [45] to examine which subnuclei were driving the group effects (code available on request). Connectivity scores were input as the dependent variable, group and condition (i.e., subnucleus) as independent variables, and age as a covariate. Post-hoc comparisons involved estimated marginal means, which adjusts for the various factors present in the linear model [46]. The Šidák correction method for investigating multiple (in our case, three) pairwise comparisons was used [47]. Results were additionally corrected for the number of significant clusters being examined for each hemisphere (0.05/2), for significance at *p*_*FWE*_ ≤ 0.03. Clusters less than 10 voxels have not been reported. Within-group subnuclei comparisons were also explored to examine connectivity differences between subnuclei for both samples.

To explore any PTSD-NEC group differences in connectivity for each individual subnuclei beyond the interaction effects identified in our primary analysis, we also conducted separate, exploratory, voxel-wise whole brain comparisons for each subnuclei in CONN. These results can be found in the Supplementary Materials.

## Results

### Sample characteristics

Groups differed in age (PTSD > NEC) but not in sex or education. Hemisphere specific differences between PTSD and NEC in subnuclei volume were only found for the left CMA (PTSD > NEC). Within each hemisphere, there were also volume differences between subnuclei (BLA > SFA > CMA). Detailed information can be found in Table 1.

### Functional connectivity analyses

**Fig. 1:**
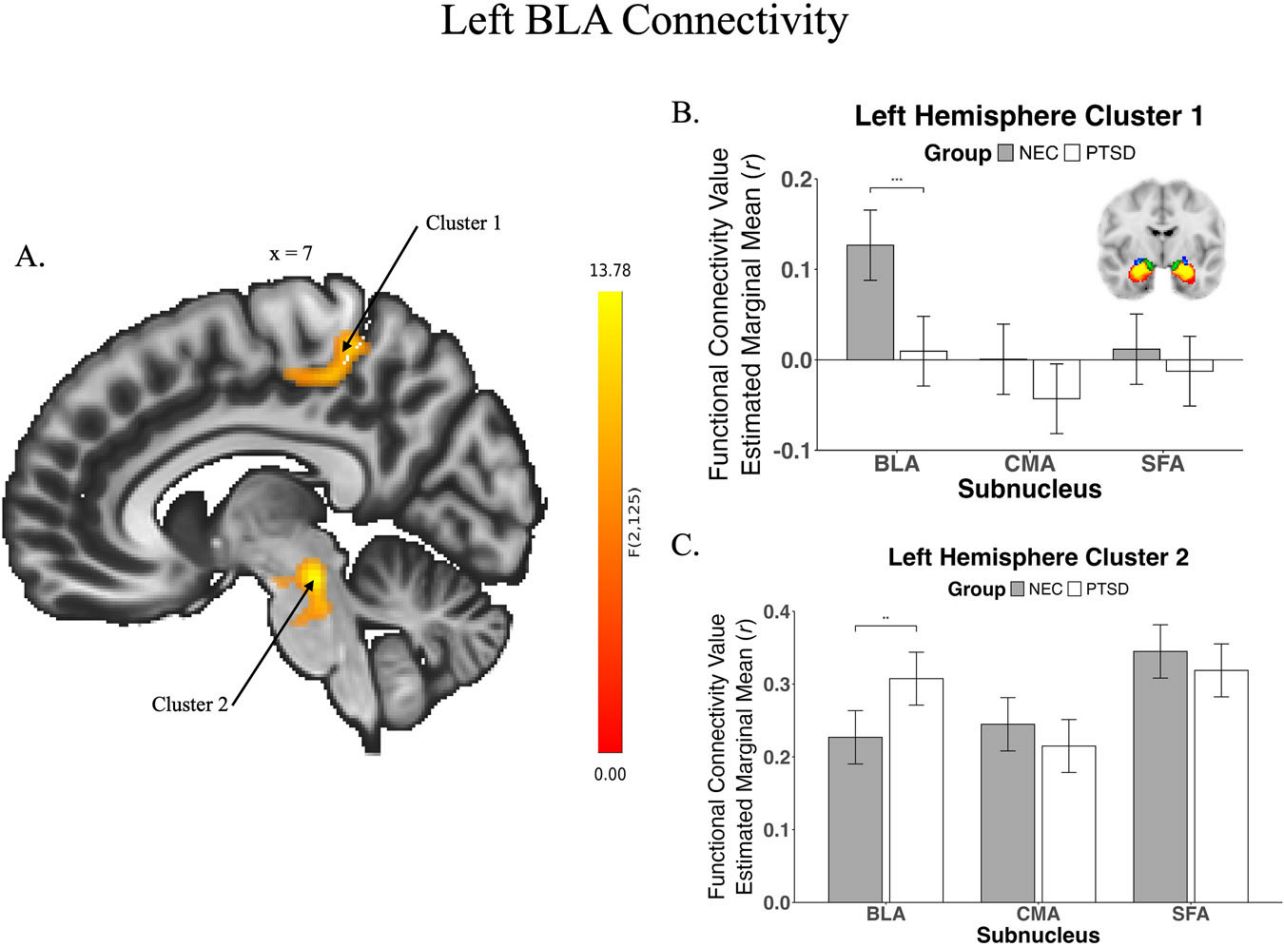
Left basolateral nucleus connectivity. Functional connectivity differences between non-trauma-exposed controls (NEC) and posttraumatic stress disorder (PTSD) for left hemisphere clusters. Image (**A**) shows significant functional connectivity differences for primarily right superior parietal areas, and for the brainstem, driven by group differences in BLA connectivity (graph (**B**): PTSD < NEC; graph (**C**): PTSD > NEC). Connectivity values were derived using voxel-wise GLM analyses, and a Threshold Free Cluster Enhancement method. Brain in image (**B**) shows percentage of overlap of amygdala subnuclei at a group level; brighter spots indicate higher overlap; Yellow/Red basolateral nuclei (BLA), Blue centromedial nuclei (CMA), Green superficial nuclei (SFA). ^**^Significant at 0.01. ^**^Significant at 0.001. Bars reflect standard error.

**Fig. 2:**
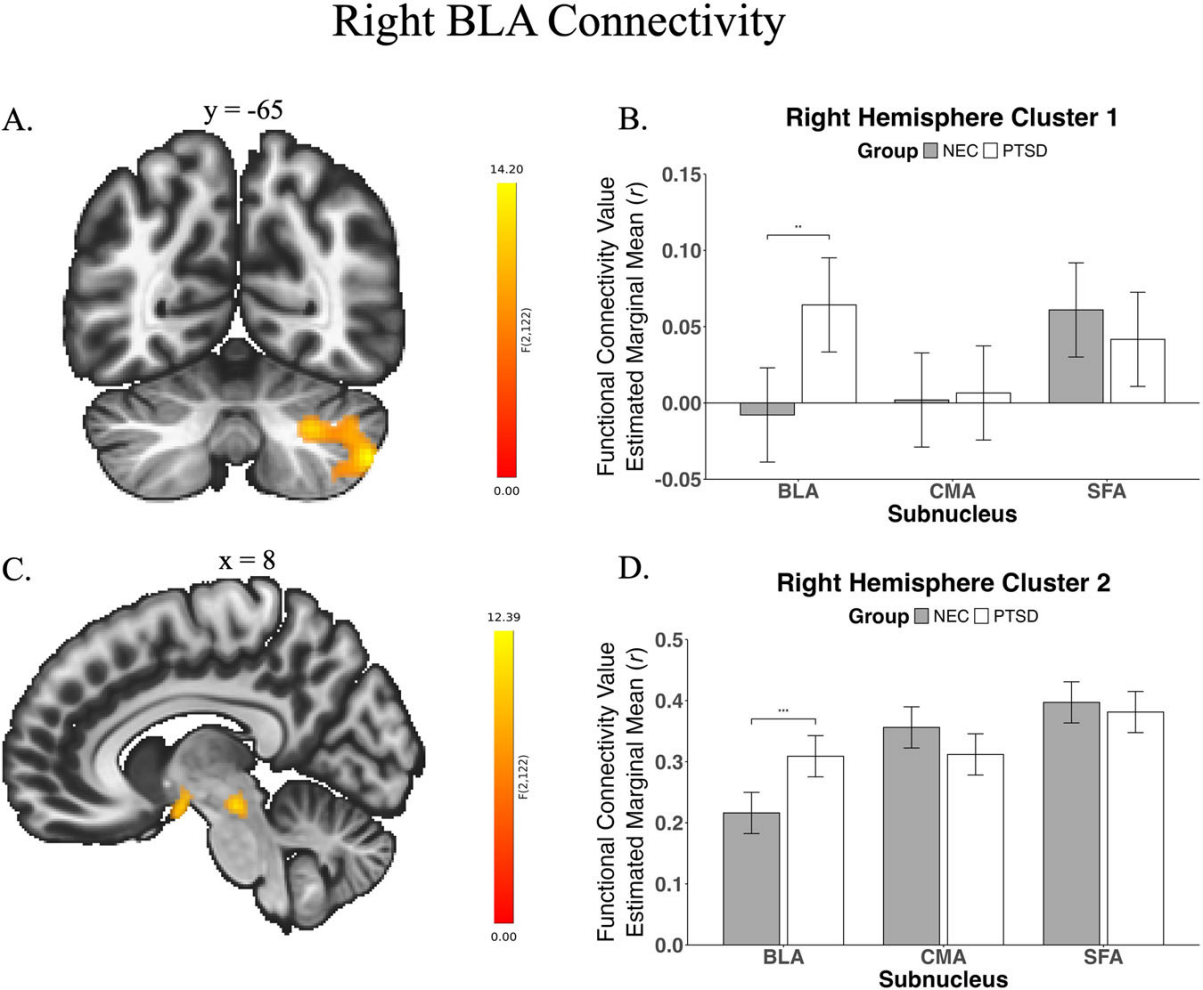
Right basolateral nucleus connectivity. Functional connectivity differences between non-trauma-exposed controls (NEC) and posttraumatic stress disorder (PTSD) for right hemisphere clusters. Images (**A**)/(**B**) show significant connectivity differences for the right cerebellum. Images (**C**)/(**D**) show significant differences for the brainstem and right pallidum. Both results for the right hemisphere were driven by differences in BLA connectivity (PTSD > NEC). Connectivity values were derived using voxel-wise GLM analyses, and a Threshold Free Cluster Enhancement method. BLA basolateral nucleus, CMA centromedial nucleus, SFA superficial nucleus. **Significant at 0.01. **Significant at 0.001. Bars reflect standard error.

Connectivity analyses showed four significant group × subnuclei interactions across left and right hemispheres (Table 2; Figs. 1 & 2). Two significant clusters were found for the left hemisphere, consisting of (1) pre/post central gyri, superior parietal lobe, precuneus, and posterior cingulate, and (2) the brain stem. Two clusters were also significant for the right hemisphere, comprising (1) the cerebellum, and (2) the brain stem and pallidum. Cluster connectivity values were extracted from CONN for further exploration of their driving factors.

**Table 2:** Significant clusters found when examining group x subnuclei differences in whole-brain functional connectivity.

Cluster	Seed	Region	Side	Complete Cluster Size	Regional Cluster Size	MNI Coordinates			*p*
				(number of voxels)		x	y	z	
Left Cluster 1	Left BLA (PTSD < NEC)	Postcentral Gyrus	R	680	307	+20	−38	+62	0.01
		Precentral Gyrus	L		71	−4	−24	+50	
		Superior Parietal Lobe	R		65	+34	−40	+60	
		Posterior Cingulate	-		65	+2	−28	+46	
		Precentral Gyrus	R		64	+6	−24	+50	
		Precuneus	-		40	+6	−40	+54	
Left Cluster 2	Left BLA (PTSD > NEC)	Brain Stem	-	341	259	+2	−28	−20	0.05
Right Cluster 1	Right BLA(PTSD > NEC)	Cerebellum Crus 2	R	789	291	+42	−64	−42	0.005
		Cerebellum Crus 1	R		224	+32	−64	−36	
		Cerebellum 8	R		122	+34	−58	−48	
		Cerebellum 7b	R		92	+38	−66	**−**50	
Right Cluster 2	Right BLA (PTSD > NEC)	Brain Stem	-	376	220	0	**−**28	**−**12	0.03
		Pallidum	R		29	+16	**−**2	**−**4	

*L* left, *R* right, *BLA* basolateral amygdala, *NEC* non-trauma-exposed controls, *PTSD* posttraumatic stress disorder.

*p* significant at a cluster threshold of *p*_FWE_ < 0.05.

Post-hoc analyses revealed that these initial interactions were driven by significant group differences in BLA connectivity for all four clusters (Table 3). The first left cluster showed higher connectivity between the BLA and cortical areas in NEC (vs PTSD); the other three showed higher connectivity between the BLA and subcortical areas in PTSD compared to NEC. Though not significant, the effects for the other subnuclei were in the opposite direction (except for the first left cluster; Figs. 1 & 2). Exploratory analyses examining whole brain voxel-wise group differences independently for individual nuclei found group differences in whole brain connectivity for the bilateral BLA and left SFA (Supplementary Materials).

**Table 3:** Post-hoc analyses examining which subnuclei comparison/s were driving group differences for significant clusters.

Cluster	Group Comparison (PTSD × NEC)		Group Means			
			NEC		PTSD	
Left Cluster 1	BLA	*t*(221) = 4.21; *p*_*FWE*_ < 0.001^*^	BLA	0.13 (0.13)	BLA:	0.007 (0.17)
	SFA	*t*(221) = 0.87; *p*_*FWE*_ = 0.76	SFA	0.01 (0.14)	SFA:	**−**0.02 (0.16)
	CMA	*t*(221) = 1.57; *p*_*FWE*_ = 0.32	CMA	0.003 (0.18)	CMA:	**−**0.05 (0.16)
Left Cluster 2	BLA	*t*(213) = **−**3.06; *p*_*FWE*_ = 0.007^*^	BLA	0.23 (0.16)	BLA:	0.31 (0.13)
	SFA	*t*(213) = 0.99; *p*_*FWE*_ = 0.69	SFA	0.35 (0.16)	SFA:	0.32 (0.16)
	CMA	*t*(213) = 1.13; *p*_*FWE*_ = 0.59	CMA	0.25 (0.14)	CMA:	0.21 (0.14)
Right Cluster 1	BLA	*t*(211) = **−**3.24; *p*_*FWE*_ = 0.004^*a^	BLA	**−**0.01 (0.13)	BLA:	0.07 (0.14)
	SFA	*t*(211) = 0.86; *p*_*FWE*_ = 0.77	SFA	0.06 (0.11)	SFA:	0.04 (0.13)
	CMA	*t*(211) = **−**0.21; *p*_*FWE*_ = 1.00	CMA	0.0001 (0.11)	CMA:	0.01 (0.12)
Right Cluster 2	BLA	*t*(216) = **−**3.82; *p*_*FWE*_ < 0.001^*^	BLA	0.22 (0.14)	BLA:	0.31 (0.14)
	SFA	*t*(216) = 0.65; *p*_*FWE*_ < 0.89	SFA	0.40 (0.14)	SFA:	0.38 (0.14)
	CMA	*t*(216) = 1.82; *p*_*FWE*_ = 0.20	CMA	0.36 (0.13)	CMA:	0.31 (0.14)

Fisher’s z correlations were used in the analysis, but raw functional connectivity correlation coefficients are reported for ease of interpretation. Šidák correction was used for three pairwise comparisons within each analysis. Results were further corrected to account for the number of clusters found to be significant for each hemisphere (0.05/2).

*NEC* non-trauma-exposed controls, *PTSD* posttraumatic stress disorder, *BLA* basolateral subnucleus, *CMA* centromedial nucleus, *SFA* superficial nucleus.

^a^This result was no longer significant when outliers were excluded from the analysis.

^*^Significant at *p*_*FWE*_ ≤ 0.03.

Due to education not differing between groups, we did not include it as a covariate in the main analysis. However, as education levels did qualitatively differ between groups, we conducted a GLM on BLA connectivity values and have included this information in the Supplementary Materials. Importantly, group differences remained robust even when controlling for any effects associated with education.

Within-group differences in subnuclei connectivity (Table 4) showed differential connectivity profiles between the BLA and SFA and all clusters in NEC (higher BLA connectivity with cortical areas; higher SFA connectivity with subcortical areas), and between the BLA and CMA and all but one cluster in the PTSD group (higher BLA connectivity with cortical and subcortical areas). Considering the high rate of comorbid GAD and MDD in the PTSD sample (28 and 49%, respectively), sensitivity analyses investigating differential subnuclei connectivity in the PTSD group controlling for these conditions were also conducted. Results were not affected (GAD: *p*’s > 0.13; MDD: *p*’s > 0.28), however, the effect of MDD (*p* = 0.02) was significant for cluster 1 of the right hemisphere (cerebellum). Sensitivity analyses were also conducted to examine any partial volume effects of amygdala subnuclei volume on connectivity results. Results were not affected (*p*’s > 0.07). Full results can be found in the Supplementary Materials.

**Table 4:** Post-hoc analyses examining subnuclei comparisons within individual groups for each significant cluster, to examine differential effects between subnuclei.

Cluster	Subnuclei Comparison			
	NEC		PTSD	
**Left Cluster 1**	**BLA-CMA**	***t*(254)** = **7.07;** ***p***_***FWE***_ < 0.001^*^	**BLA-CMA**	***t*(254)** = **2.97;** ***p***_***FWE***_ = 0.01^*^
	**BLA-SFA**	***t*(254)** = **6.45;** ***p***_***FWE***_ < 0.001^*^	BLA-SFA	*t*(254) = 1.25; *p*_*FWE*_ = 0.51
	CMA-SFA	*t*(254) = **−**0.63; *p*_*FWE*_ = 0.90	CMA-SFA	*t*(254) = **−**1.72; *p*_*FWE*_ = 0.24
**Left Cluster 2**	BLA-CMA	*t*(254) = **−**1.10; *p*_*FWE*_ = 0.62	**BLA-CMA**	***t*(254)** = **5.74;** ***p***_***FWE***_ < 0.001^*^
	**BLA-SFA**	***t*(254)** = **−7.27;** ***p***_***FWE***_ < 0.001^*^	BLA-SFA	*t*(254) = −0.70; *p*_*FWE*_ = 0.86
	CMA-SFA	*t*(254) = −6.17; *p*_*FWE*_ < 0.001^*^	CMA-SFA	*t*(254) = −6.45; *p*_*FWE*_ < 0.001^*^
**Right Cluster 1**	BLA-CMA	*t*(248) = **−**0.07; *p*_*FWE*_ = 0.86	**BLA-CMA**	***t*(248)** = **4.17;** ***p***_***FWE***_ < 0.001^*^
	**BLA-SFA**	***t*(248)** = **−4.97**; ***p***_***FWE***_ < 0.001^*^	BLA-SFA	*t*(248) = 1.63; *p*_*FWE*_ = 0.28
	CMA-SFA	*t*(248) = −4.26; *p*_*FWE*_ < 0.001^*^	CMA-SFA	*t*(248) = −2.54; *p*_*FWE*_ = 0.04
**Right Cluster 2**	**BLA-CMA**	***t*(248)** = **−9.05;** ***p***_***FWE***_ < 0.001^*^	BLA-CMA	*t*(248) = −0.19; *p*_*FWE*_ = 1.00
	**BLA-SFA**	***t*(248)** = **−11.7;** ***p***_***FWE***_ < 0.001^*^	**BLA-SFA**	***t*(248)** = **−4.68;** ***p***_***FWE***_ < 0.001^*^
	CMA-SFA	*t*(248) = −2.65; *p*_*FWE*_ = 0.03^*^	CMA-SFA	*t*(248) = −4.49; *p*_*FWE*_ < 0.001^*^

Šidák correction was used for three pairwise comparisons within each analysis, with an extra family-wise error correction for the number of significant clusters in each hemisphere (0.05/2). Results in **bold** signify significant group effects in previous linear model.

*NEC* non-trauma-exposed controls, *PTSD* posttraumatic stress disorder, *BLA* basolateral subnucleus, *CMA* centromedial nucleus, *SFA* superficial nucleus.

^*^Significant at *p*_*FWE*_ ≤ 0.03.

## Discussion

Our study sought to examine differences in task-derived intrinsic functional connectivity of amygdala subnuclei in individuals with PTSD relative to non-trauma-exposed controls (NEC). A group by subnuclei interaction was found for two clusters in both left and right hemispheres. When probed further, this interaction was found to be driven by significant group differences in basolateral (BLA) connectivity with areas involved in reward, sensorimotor, and self-referential processing [6, 48, 49]. Specifically, *decreased* connectivity between the left BLA and cortical sensorimotor/self-referential processing areas—right superior parietal lobe, bilateral precentral gyri, right postcentral gyrus, and precuneus and posterior cingulate—was found in PTSD relative to NEC. In contrast, *increased* connectivity in PTSD between the left BLA and the brainstem, and the right BLA and subcortical areas including the brainstem, cerebellum, and pallidum were also found—implicating dysfunctions in brain areas involved in arousal, salience, sensory, threat, and reward processing [48–50]. These results are in line with prior literature and suggest that subject-specific delineation of amygdala subnuclei is a useful and reliable way to probe region-specific differences in intrinsic functional in PTSD.

Driving the group by subnuclei interaction for one left hemisphere cluster was decreased connectivity in PTSD between the left BLA and areas comprising the bilateral pre-central gyri, right postcentral gyri and superior parietal lobe, and the precuneus and posterior cingulate. The direction of this effect was similar for the CMA and SFA but was not significant. The pre- and postcentral gyri encompass the primary motor cortices and somatosensory cortices, respectively, and support emotion-action interaction and emotion regulation [51, 52]. Located adjacently, the superior parietal lobe, which is part of the cognitive control network and implicated in voluntary attentional shifts for different informational domains (e.g., perceptions, memory, etc.; [53]), also has strong connections to sensorimotor networks [54]. Electrophysiological studies in non-human primates have shown somatosensory stimulation activates the amygdala [55], while diffusion tensor imaging studies in humans have found the BLA and SFA to be connected to the pre- and postcentral gyri [56]. Structurally, lower cortical thickness for the superior parietal lobes has also been found in PTSD [57] and gray matter volume of the left superior parietal lobe has been found to be correlated with anxiety in chronic PTSD [58]. One study found higher functional connectivity between a bilateral amygdala seed and these same areas in the left hemisphere for trauma-related (vs neutral) images for recently trauma-exposed (vs non-trauma-exposed) individuals [59]. In this context it is interesting to note that greater negative functional connectivity between the left amygdala and left precentral gyrus at rest has been found to be a predictor of greater PTSD symptom severity at six months [60]. Of note, although connectivity differences with sensorimotor areas in task-derived functional connectivity could be a residual task effect due to intrinsic connectivity derived from tasks, our results do show concordance with studies in a previous review on resting-state functional connectivity of subnuclei in PTSD [9], suggesting that this difference is at the intrinsic level rather than due to tasks.

The other two regions included in this cluster were the precuneus and posterior cingulate, which are both part of the DMN and involved in self-referential processing [61]. Decreased coupling between the areas involved in the DMN has been observed in PTSD at rest [4, 62], but increased coupling between the amygdala and areas of the DMN has also been seen for task-related paradigms [63]. In accordance, greater precuneus-amygdala connectivity has been observed in NEC when focusing on unpleasant parts of an image [64], and hyperactivation of these areas in PTSD has also been found in symptom provocation studies [18], suggesting this pathway is an active part of negative attentional control processes. Additionally, neurofeedback downregulating the posterior cingulate has been associated with decreases in amygdala connectivity in PTSD [65], suggesting a role for the posterior cingulate in PTSD that may involve modulation of the amygdala, particularly the BLA. However, increased, task-related coupling between these areas of the DMN and the BLA may also indicate task-related attenuation of signal [66]. Nevertheless, this still suggests that the areas are connected, and that they may be contributing to the neural signature underlying PTSD.

The connectivity between the amygdala and the pre- and postcentral gyri is thought to underlie emotional modulation of subjective sensory experience [67]. If such modulation shapes these self-referential processes associated with the precuneus and posterior cingulate in a non-trauma-exposed brain, this modulation may become dysfunctional in PTSD through a disconnect between these areas. Such a dysfunction may even underlie intrusive-like reexperiencing symptomatology that is characteristic of PTSD; however, this will require future research.

In contrast, the other three clusters were driven by increased connectivity with the BLA in PTSD; though not significant, effects for the CMA and SFA were trending in the opposite direction. Significantly increased connectivity in PTSD was found between the bilateral BLA and the brainstem, an area of the brain comprising various nuclei involved in vital processes such as arousal regulation, breathing, and consciousness, as well as affective and stress responses [68, 69]. The brainstem receives both interoceptive and exteroceptive raw sensory projections, including extreme arousal fluctuations such as those invoked by traumatic stress [48]. The BLA itself receives information about unconditioned stimuli from the brainstem [70] and projects to the brainstem via the central nucleus [15], allowing it to associate stimuli with valence [71], and to shape behavioral responses towards these associations. More specifically, connectivity between the locus coeruleus of the brainstem and the BLA is required for threat conditioning and extinction learning [72, 73]. Periaqueductal gray brainstem responses to fear, that are usually contained by inhibitory processes, are also thought to be disinhibited in PTSD, allowing for the constant transmission of aversive information to higher levels structures like the amygdala [74]. This might explain our finding of greater intrinsic functional connectivity between these areas in PTSD and accords with the role of the BLA in goal directed escape in humans and in rodents [75]. Under imminent and unescapable threat, the BLA in both humans and rodents is essential for the selection and execution of rapid escape behavior through a BLA → central nucleus → brainstem pathway, and plays a role in switching between passive defense and active escape in rodents [76]. A task-based study found higher left BLA-brainstem connectivity for trauma-related images compared to neutral images in PTSD [77]. Taken together, this may indicate that an increase in connectivity between the BLA and brainstem is a general connectivity profile in PTSD that facilitates and maintains arousal-driven, bottom-up salience detection of threatening stimuli, contributing to dysfunctional fear-learning and extinction processes.

Significantly increased connectivity that was also found to be driving the interaction effect, was found for the PTSD group between the right BLA and right pallidum. The pallidum is considered a key hub for brain circuitry involved in reward processing, hedonic, and motivational behavior [78], and in the regulation of adaptive behavior and social reward, both of which are affected during severe stress [79]. Preclinical rodent work has found evidence for a pathway between the BLA and pallidum that involves reward wanting [72]. Work in non-human primates has found this pathway plays a role in the encoding of aversive contextual information for avoidance behavior [80]. Preclinical studies in mice have found that the ventral pallidum projects directly to the BLA (but receives projections from the CMA), perhaps shedding some light on the directionality of our finding; however, this interpretation would have to be further explored using techniques such as dynamic causal modelling [81]. Nevertheless, with evidence of reward learning deficits during reward anticipation in PTSD [49], the higher functional connectivity we found between the BLA and pallidum might contribute to this dysfunction in reward-seeking behavior.

Connectivity between the right BLA and right cerebellum was also greater in PTSD and found to be a driver of the group by subnuclei interaction. While the cerebellum is known to be involved in motor control, it has been shown to play a role in memory, in sensory processing, attention, social, and emotion processes [82, 83], and in conditioning and extinction processes in rodents and mice [3, 84]. The areas that showed significant connectivity in our study—Crus 1, Crus 2, and area 7b, have all been found to be involved in task-unfocused non-motor representation [50]. Furthermore, areas 7 and 8 in humans also show evidence of functional connectivity with the amygdala [85]. Preclinical work in mice has found that cerebellar output nuclei have disynaptic connectivity with the BLA through certain thalamic nuclei, a connection which may represent information about salience or valence of stimuli [86]. Although work on the cerebellum in PTSD is limited, smaller cerebellum volume has been found to characterize PTSD relative to both NEC and trauma-exposed controls, and to be associated with greater symptom severity [87, 88]. However, no specific amygdala-cerebellum connectivity was found in a study that sought to characterize the functional connectivity of the cerebellum in PTSD [89]. Nevertheless, greater connectivity between the right BLA and right cerebellum in PTSD may represent an aberrant salience network at rest, a neural signature that facilitates dysfunctional switching between the salience and default mode networks in PTSD. And while there is evidence for the dysfunctional interaction between the default mode network and the salience network in PTSD [62], the neurobiological contributions to this dysfunction require further exploration.

Additionally, it is worth noting the lack of findings for the CMA and SFA. It is possible that the lack of effects could be due to smaller sizes of these substructures and that the functional signal-to-noise ratio from the larger BLA structure provided more statistical power. Another perspective is that a task driven paradigm might offer greater detectability of these effects in PTSD. The role of the CMA lies primarily in response generation [12] which might lend itself better to task paradigms for any discernible group differences. Indeed, stimulation of the central nucleus in rabbits increases both attention and orienting responses [90], and in primates, the CMA has also been found to play a role in allocating attention to salient stimuli and in initiating appropriate autonomic responses to task events [91]. In humans, higher CMA activity has also been found to be related to allocating salience and responses in a gustatory task [92]. By their nature, such task-relevant connectivity patterns of the CMA are likely unable to be reliably detected during either task-derived or intrinsic resting-state activity; though it should be noted that four of nine studies in a recent review of resting-state fMRI amygdala subnuclei functional connectivity in PTSD did find differential CMA connectivity between groups. However, two studies used a 1.5 T scanner [93, 94], and one study only found the result for their subgroup of males [20], which could potentially account for our null findings. The SFA, in contrast, is mostly known for its role in olfactory processing [11]. However, in humans, it has been found to extract the social value of sensory information and to use social processing and emotion regulation to inform behavior [17]. Compared to the BLA and CMA, the SFA in humans shows more activation to facial expressions [17], and is more connected to the occipital lobe when experiencing acute stress [95]. Only two studies have examined SFA functional connectivity in PTSD at rest [19, 96], but only one of those reported a significant result (albeit in a sample of 20 participants). Interestingly, in a larger sample of 50 participants, group differences in SFA connectivity were found for patients with major depression without anxiety; but for those with anxiety, only patients were found to have lower BLA and SFA activity [97]. Collectively, this might suggest that group differences in SFA connectivity are possibly only detectable during task paradigms (as with the CMA); or, that differential SFA activity is only evident within groups—which was evident in our sample, albeit primarily for the NEC group. In any case, the connectivity patterns of both the CMA and SFA require future research to investigate their contribution to the neurobiology of PTSD.

Finally, some limitations inherent to this study should be considered. There are a number of technical issues when imaging the subnuclei of the amygdala [36]. These are largely due to the location of subcortical structures which results in lower fMRI signal-to-noise ratio [98] and to the boundaries subcortical structures share with air and bone which makes them more prone to signal loss due to magnetic field inhomogeneities [99]. This makes the delineation of functional connectivity of small structures like the amygdala challenging, especially at the relatively poor resolution of 3T fMRI. This limitation indicates the potential utility of using ultra-high field MRI for answering these questions, as it produces more detailed images which allow for a more accurate delineation of subnuclei and their connectivity profiles. Additionally, although we did not delineate the hippocampus, brainstem, and cerebellum as we did with the amygdala, finer parcellation of these brain areas may also provide a more nuanced picture of their functional heterogeneity and relative connectivity profiles with amygdala subnuclei. A second consideration pertains to the sample used in this study. Though the sample size was sufficiently large, our use of a non-trauma control group as a comparison cannot determine if these results are specific to PTSD or due to trauma-exposure in general. Due to the paucity of research in the area, it is important to examine any differences in amygdala subnuclei neural circuitry in PTSD. Such results are then able to be probed by further research and the inclusion of a trauma-exposed control cohort to understand these differences in relation to trauma-exposure and PTSD in general. A final limitation lies in the nature of intrinsic functional connectivity, which examines brain activity at rest as opposed to during a task. Intrinsic functional connectivity derived from block designs seems to map onto pure resting-state data more accurately than event-related designs (which comprised three of our five tasks; [25]). Yet data from event-related residuals remains qualitatively similar to resting-state data, even though they have quantitatively distinct properties. Though this may not be the most optimal way of delineating functional connectivity differences between amygdala subnuclei, considering the available evidence supporting amygdala hyperconnectivity to subcortical areas after trauma (outlined above), such results remain useful but should nevertheless be interpreted with caution.

This study sought to examine group differences between PTSD and NEC in intrinsic functional connectivity profiles of amygdala subnuclei to better characterize the underlying neurobiology of PTSD. We found significant group by subnuclei interactions in the connectivity profiles of left hemisphere and right hemisphere subnuclei, driven by group differences in BLA connectivity. Specifically, individuals with PTSD showed lower BLA connectivity to cortical areas involved in sensorimotor and self-referential processing (pre/postcentral gyri, superior parietal lobe, precuneus, posterior cingulate), but higher BLA connectivity to the subcortical brainstem, cerebellum, and pallidum—areas involved in sensory processing, task-unfocused representation, and reward processing. These results largely accord with PTSD models [100], and together suggest that the BLA is a key driver of the intrinsic brain dysfunction in PTSD that pertains to arousal, reward, and self-referential processing.

## Supplementary information

Supplementary Material

## Data Availability

Data for this project is not publicly available, however, can be provided by the authors upon reasonable request.
