# Supplementary material for: Functional connectivity profiles of amygdala subregions in posttraumatic stress disorder
Source: Transl Psychiatry. 2025 Aug 14;15:280. doi: 10.1038/s41398-025-03508-y (PMC12350740; doi:10.1038/s41398-025-03508-y)
Supplement: Supplementary file 1 — Supplementary Material [file 41398_2025_3508_MOESM1_ESM.docx]

# **Supplementary Information**

# **Methods**

Additional linear mixed effects models were conducted to examine the effect of education on connectivity values: although education did not quantitatively differ between groups it did show a qualitative difference, with a higher number of PTSD participants having lower levels and higher NEC participants having higher levels of schooling. Average timeseries correlations from all four significant clusters extracted from CONN were put into separate linear mixed effects models in R. The effect of interest (group x subnuclei interaction) was examined, with connectivity values as the dependent variable, and education as a covariate. Education values reported as “Other” or “NA” were removed from analysis.

Additional exploratory voxel-wise analyses were also conducted to examine group differences between subnuclei that might not be evident when including all three subnuclei in the main seed-to-voxel analysis. Methods from the main analysis were replicated, this time only investigating group effects for each individual subnuclei (family-wise error-corrected *p*-FDR<.05). Results were additionally corrected for the number of significant clusters being examined for each subnucleus, for significance at *p*_FWE_≤.05 (one significant cluster) or *p*_FWE_≤.03 (two significant clusters).

# **Results**

Linear mixed effects models for the left hemisphere revealed no significant effects of education on connectivity values (*p*’s>.08), with group x subnuclei interactions remaining significant predictors of differences in functional connectivity (Tables/Figures S1 & S2). While models for the right hemisphere did reveal significant cubic effects of education on subnuclei connectivity, group x subnuclei interactions remained significant (Tables/Figures S3 & S4). Effects showed the same pattern across groups.

**Table S1.** Linear mixed effects modelling group x subnuclei interaction effect on amygdala functional connectivity for left hemisphere Cluster 1, controlling for education.

|  | **Connectivity** | | |
| --- | --- | --- | --- |
| *Predictors* | *Estimates* | *CI* | *p* |
| (Intercept) | 0.10 | 0.04 – 0.15 | **0.001** |
| Group [PTSD] | -0.10 | -0.16 – -0.04 | **0.001** |
| ROI [CMA.L] | -0.12 | -0.16 – -0.09 | **<0.001** |
| ROI [SFA.L] | -0.12 | -0.15 – -0.08 | **<0.001** |
| Education [linear] | 0.04 | -0.04 – 0.13 | 0.326 |
| Education [quadratic] | 0.04 | -0.03 – 0.11 | 0.259 |
| Education [cubic] | -0.11 | -0.23 – 0.02 | 0.087 |
| Education [4th degree] | 0.02 | -0.08 – 0.11 | 0.715 |
| Group [PTSD] × ROI [CMA.L] | 0.06 | 0.01 – 0.11 | **0.025** |
| Group [PTSD] × ROI [SFA.L] | 0.09 | 0.04 – 0.14 | **0.001** |
| **Random Effects** | | | |
| σ^2^ | 0.01 | | |
| τ_00_ _ID_ | 0.01 | | |
| ICC | 0.58 | | |
| N _ID_ | 120 | | |
| Observations | 360 | | |
| Marginal R^2^ / Conditional R^2^ | 0.141 / 0.643 | | |

**Table S2.** Linear mixed effects modelling group x subnuclei interaction effect on amygdala functional connectivity for left hemisphere Cluster 2, controlling for education.

|  | **Connectivity** | | |
| --- | --- | --- | --- |
| *Predictors* | *Estimates* | *CI* | *p* |
| (Intercept) | 0.25 | 0.20 – 0.31 | **<0.001** |
| Group [PTSD] | 0.08 | 0.02 – 0.14 | **0.006** |
| ROI [CMA.L] | 0.02 | -0.02 – 0.05 | 0.372 |
| ROI [SFA.L] | 0.12 | 0.09 – 0.15 | **<0.001** |
| Education [linear] | -0.02 | -0.10 – 0.06 | 0.663 |
| Education [quadratic] | -0.04 | -0.11 – 0.02 | 0.216 |
| Education [cubic] | 0.11 | -0.01 – 0.22 | 0.080 |
| Education [4th degree] | -0.04 | -0.14 – 0.05 | 0.339 |
| Group [PTSD] × ROI [CMA.L] | -0.10 | -0.15 – -0.06 | **<0.001** |
| Group [PTSD] × ROI [SFA.L] | -0.10 | -0.15 – -0.06 | **<0.001** |
| **Random Effects** | | | |
| σ^2^ | 0.01 | | |
| τ_00_ _ID_ | 0.01 | | |
| ICC | 0.61 | | |
| N _ID_ | 120 | | |
| Observations | 360 | | |
| Marginal R^2^ / Conditional R^2^ | 0.122 / 0.658 | | |


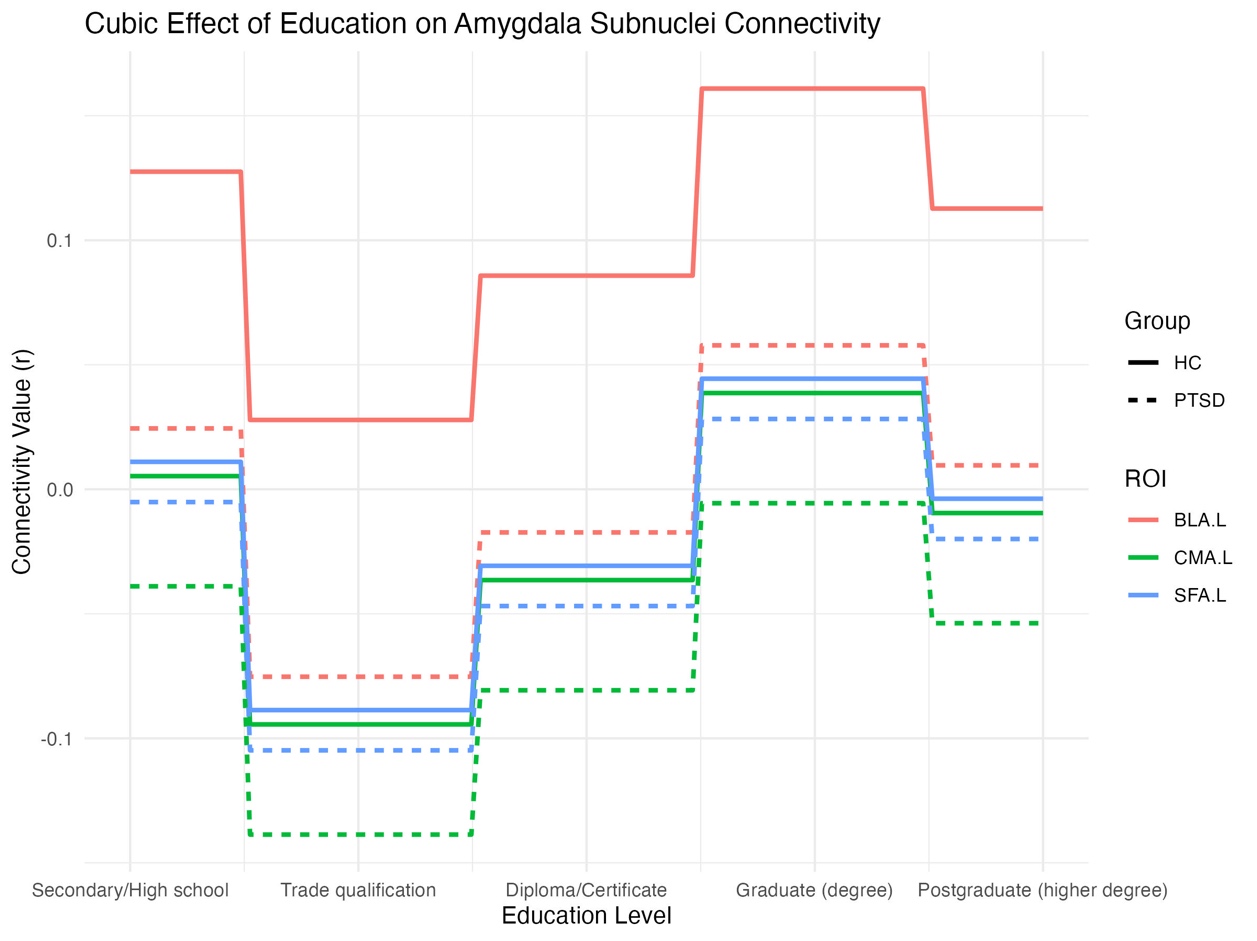


*Figure S1.* Non-significant effects (*p*=.08) of education on functional connectivity of left hemisphere amygdala subnuclei and Cluster 1. Connectivity values are Fisher’s *z* bivariate correlations. HC Healthy Controls (no trauma exposure). PTSD Posttraumatic Stress Disorder. ROI Region of Interest. BLA.L Left Basolateral Amygdala Subnucleus. CMA.L Left Centromedial Amygdala Subnucleus . SFA.L Left Superficial Amygdala Subnucleus.


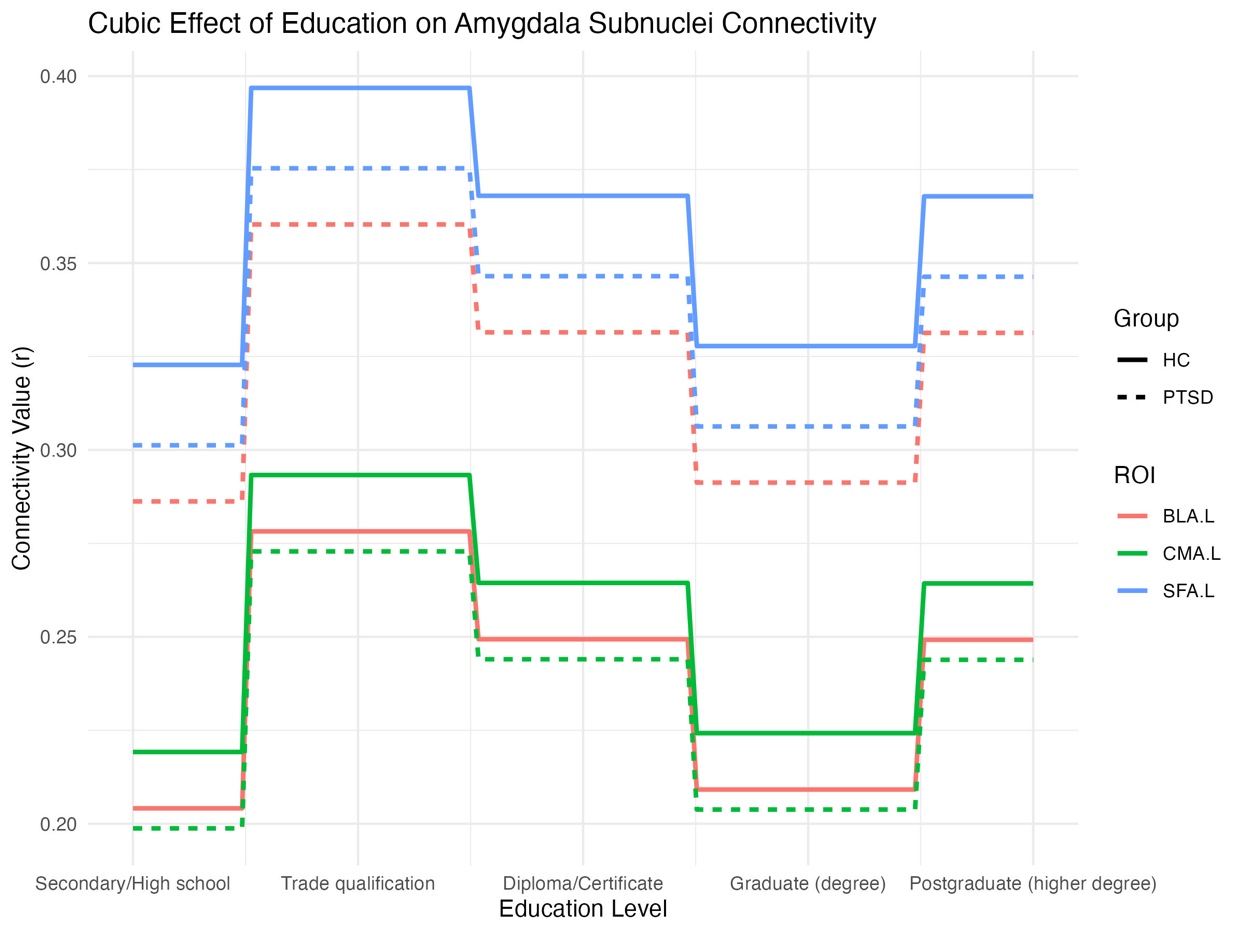


*Figure S2.* Non-significant effects (*p*=.08) of education on functional connectivity of left hemisphere amygdala subnuclei and Cluster 2. Connectivity values are Fisher’s *z* bivariate correlations. HC Healthy Controls (no trauma exposure). PTSD Posttraumatic Stress Disorder. ROI Region of Interest. BLA.L Left Basolateral Amygdala Subnucleus. CMA.L Left Centromedial Amygdala Subnucleus . SFA.L Left Superficial Amygdala Subnucleus.

**Table S3.** Linear mixed effects modelling group x subnuclei interaction effect on amygdala functional connectivity for right hemisphere Cluster 1, controlling for education.

|  | **Connectivity** | | |
| --- | --- | --- | --- |
| *Predictors* | *Estimates* | *CI* | *p* |
| (Intercept) | 0.04 | -0.01 – 0.08 | 0.093 |
| Group [PTSD] | 0.04 | -0.00 – 0.09 | 0.066 |
| ROI [CMA.R] | 0.01 | -0.02 – 0.04 | 0.475 |
| ROI [SFA.R] | 0.07 | 0.04 – 0.10 | **<0.001** |
| Education [linear] | -0.08 | -0.14 – -0.01 | **0.023** |
| Education [quadratic] | -0.02 | -0.08 – 0.03 | 0.388 |
| Education [cubic] | 0.10 | 0.01 – 0.20 | **0.033** |
| Education [4th degree] | -0.04 | -0.12 – 0.03 | 0.275 |
| Group [PTSD] × ROI [CMA.R] | -0.07 | -0.11 – -0.03 | **0.001** |
| Group [PTSD] × ROI [SFA.R] | -0.08 | -0.13 – -0.04 | **<0.001** |
| **Random Effects** | | | |
| σ^2^ | 0.01 | | |
| τ_00_ _ID_ | 0.01 | | |
| ICC | 0.58 | | |
| N _ID_ | 117 | | |
| Observations | 351 | | |
| Marginal R^2^ / Conditional R^2^ | 0.097 / 0.624 | | |

**Table S4.** Linear mixed effects modelling group x subnuclei interaction effect on amygdala functional connectivity for right hemisphere Cluster 2, controlling for education.

|  | **Connectivity** | | |
| --- | --- | --- | --- |
| *Predictors* | *Estimates* | *CI* | *p* |
| (Intercept) | 0.25 | 0.21 – 0.30 | **<0.001** |
| Group [PTSD] | 0.09 | 0.04 – 0.15 | **<0.001** |
| ROI [CMA.R] | 0.14 | 0.11 – 0.17 | **<0.001** |
| ROI [SFA.R] | 0.18 | 0.15 – 0.21 | **<0.001** |
| Education [linear] | -0.05 | -0.12 – 0.03 | 0.213 |
| Education [quadratic] | -0.04 | -0.10 – 0.02 | 0.230 |
| Education [cubic] | 0.13 | 0.02 – 0.24 | **0.016** |
| Education [4th degree] | -0.10 | -0.18 – -0.02 | **0.020** |
| Group [PTSD] × ROI [CMA.R] | -0.14 | -0.18 – -0.10 | **<0.001** |
| Group [PTSD] × ROI [SFA.R] | -0.11 | -0.15 – -0.06 | **<0.001** |
| **Random Effects** | | | |
| σ^2^ | 0.01 | | |
| τ_00_ _ID_ | 0.01 | | |
| ICC | 0.60 | | |
| N _ID_ | 117 | | |
| Observations | 351 | | |
| Marginal R^2^ / Conditional R^2^ | 0.200 / 0.682 | | |


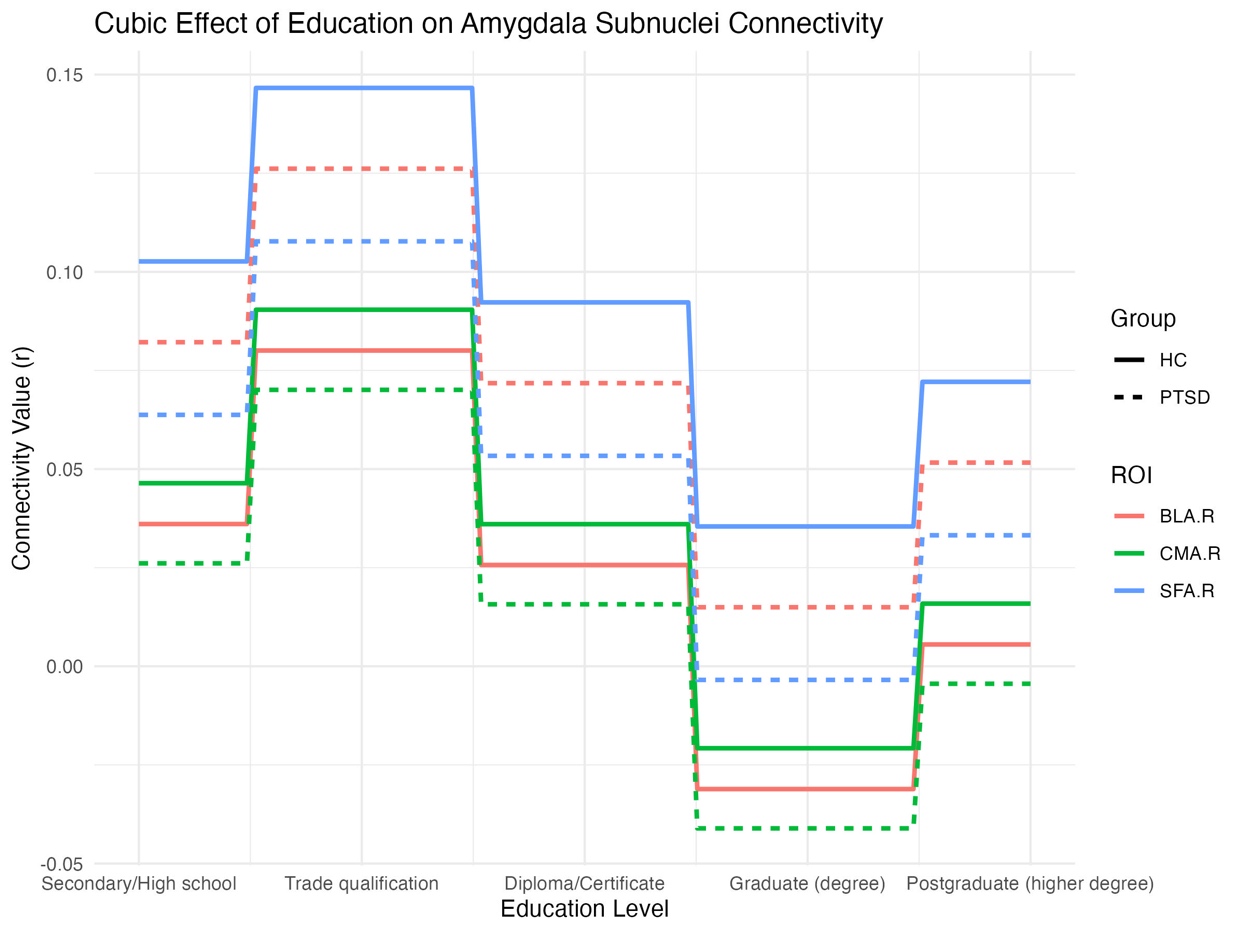


*Figure S3.* Significant effects (*p*=.03) of education on functional connectivity of right hemisphere amygdala subnuclei and Cluster 1. Group x Subnuclei effect remained significant. Connectivity values are Fisher’s *z* bivariate correlations. HC Healthy Controls (no trauma exposure). PTSD Posttraumatic Stress Disorder. ROI Region of Interest. BLA.R Right Basolateral Amygdala Subnucleus. CMA.R Right Centromedial Amygdala Subnucleus . SFA.R Right Superficial Amygdala Subnucleus.


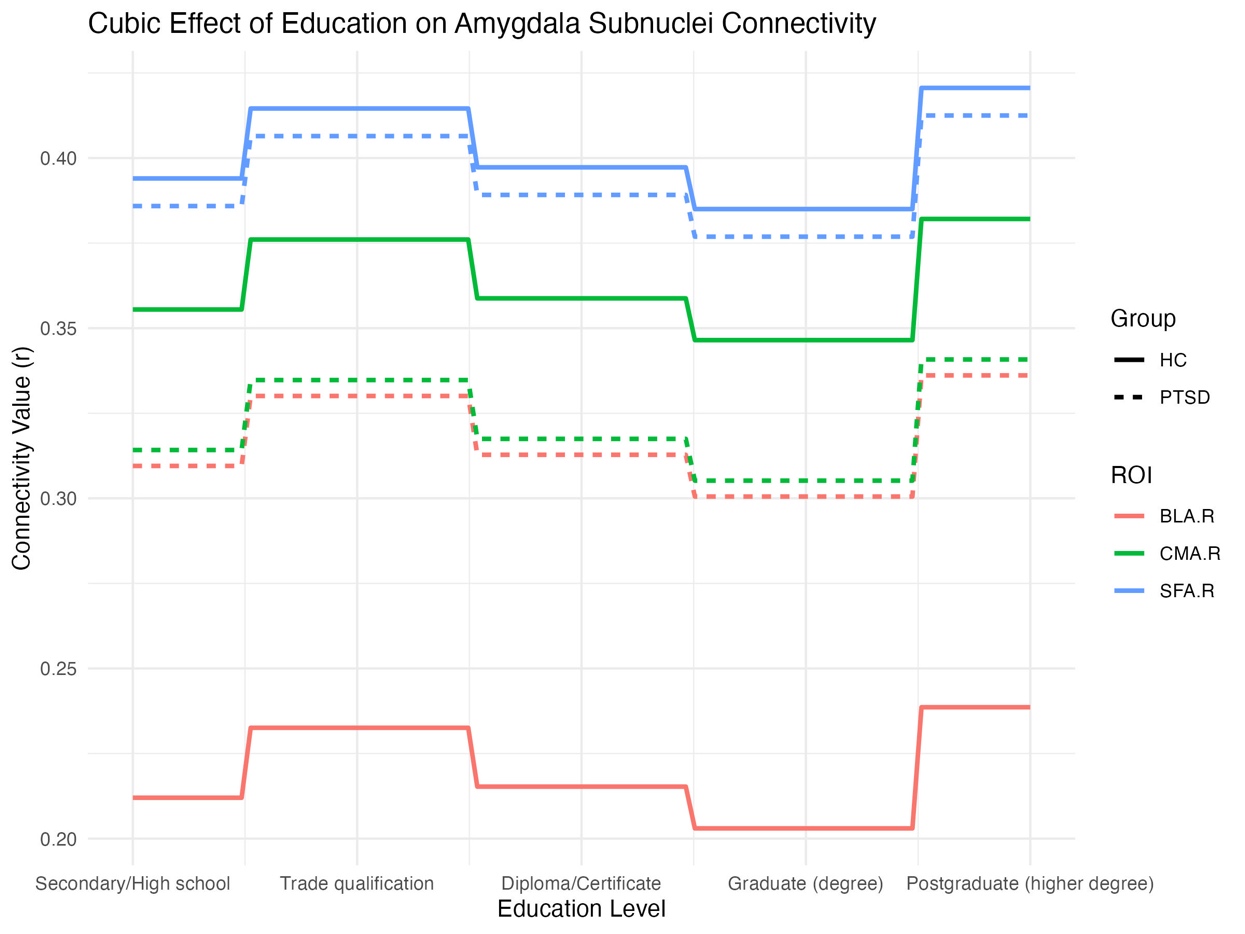


*Figure S4.* Significant effects (*p*=.02) of education on functional connectivity of right hemisphere amygdala subnuclei and Cluster 2. Group x Subnuclei effect remained significant. Connectivity values are Fisher’s *z* bivariate correlations. HC Healthy Controls (no trauma exposure). PTSD Posttraumatic Stress Disorder. ROI Region of Interest. BLA.R Right Basolateral Amygdala Subnucleus. CMA.R Right Centromedial Amygdala Subnucleus . SFA.R Right Superficial Amygdala Subnucleus.

Connectivity analyses revealed group differences for bilateral BLA and the left SFA, with the left BLA showing the strongest effects (Table S5). The left BLA showed significantly lower connectivity in PTSD with superior frontal and parietal areas, the precuneus, anterior, and posterior cingulate cortices (*t*(126)=-5.17; *p*<.001; EMM (SE): NEC=.12 (.02); PTSD=-.003 (.02)). In contrast, this region also showed higher connectivity in PTSD with subcortical areas and the frontal pole/orbital cortex (*t*(126)=5.07; *p*<.001; EMM (SE): NEC=.06 (.02); PTSD=.19 (.02)). The left SFA showed lower connectivity in PTSD with left temporal areas (*t*(126)=-6.28; *p*<.001; EMM (SE): NEC=.19 (.02); PTSD=.05 (.02)). As with the left BLA, the right BLA also showed higher connectivity in PTSD with the thalamus and pallidum (*t*(123)=4.83; *p*<.001; EMM (SE): NEC=.03 (.02); PTSD=.17 (.02)).

Sensitivity analyses were conducted to investigate differential connectivity in the PTSD group controlling for comorbid generalized anxiety (GAD; 28% of participants) and major depression (MDD; 49% of participants). Separate linear mixed models were conducted for these conditions, including GAD and MDD as covariates (Model: Connectivity Value = ROI (BLA/CMA/SFA) + MDD (or GAD)). Generalised anxiety did not have any effect on connectivity results (*p*’s>.05 for all models; Tables S6-S9). The effect of MDD was only significant for Cluster 2 for the right hemisphere (including the cerebellum; p’s>.02; Tables S10-S13).

Additional sensitivity analyses were also conducted to examine any partial volume effects of amygdala subnuclei volume on functional connectivity results. Separate linear mixed models were conducted for each significant cluster (e.g., Connectivity Value = Group (HC/PTSD) * ROI (left BLA/CMA/SFA) + Age + Volume (left BLA/CMA/SFA) + (1 | Participant). Amygdala subnuclei volumes were not found to affect the existing interaction results (*p*’s>.05 for all models; Tables S14-S17).

**Table S5.** Significant group differences for functional connectivity of individual subnuclei with the whole brain.

| **Seed** | **Region** | **Side** | **Complete Cluster Size** | **Regional Cluster Size** | ***p*** | **MNI Coordinates** | | |
| --- | --- | --- | --- | --- | --- | --- | --- | --- |
|  |  |  | **(number of voxels)** | |  | **x** | **y** | **z** |
| Left BLA | Postcentral Gyrus | R | 4296 | 607 | <.001^**^  PTSD < NEC | +26 | -30 | +58 |
|  | Precentral Gyrus | R |  | 573 |  | +20 | -22 | +60 |
|  | Precentral Gyrus | L |  | 481 |  | -16 | -24 | +62 |
|  | Precuneus | - |  | 409 |  | -4 | -44 | +52 |
|  | Postcentral Gyrus | L |  | 245 |  | -20 | -40 | +58 |
|  | Posterior Cingulate | - |  | 173 |  | -2 | -26 | +44 |
|  | Superior Parietal Lobe | R |  | 103 |  | +22 | -46 | +58 |
|  | Anterior Cingulate Gyrus | - |  | 56 |  | +2 | -12 | +40 |
|  | Superior Parietal Lobe | L |  | 47 |  | -20 | -48 | +60 |
| Left BLA | Putamen | R | 791 | 116 | .01^**^  PTSD > NEC | +18 | +10 | -4 |
|  | Thalamus | L |  | 104 |  | -8 | -8 | +4 |
|  | Pallidum | L |  | 86 |  | -16 | -4 | -2 |
|  | Pallidum | R |  | 68 |  | +16 | +2 | -2 |
|  | Thalamus | R |  | 49 |  | +6 | -6 | +4 |
|  | Frontal Pole | R |  | 25 |  | +32 | +36 | -8 |
|  | Frontal Orbital Cortex | R |  | 15 |  | +34 | +30 | -6 |
| Left SFA | Anterior Temporal Fusiform Cortex | L | 499 | 160 | .03^*^  PTSD < NEC | -30 | -4 | -44 |
|  | Temporal Pole | L |  | 129 |  | -34 | +12 | -44 |
|  | Anterior Parahippocampal Gyrus | L |  | 104 |  | -18 | -6 | -32 |
| Right BLA | Thalamus | L | 451 | 185 | .04^*^  PTSD > NEC | -6 | -8 | +4 |
|  | Pallidum | L |  | 94 |  | -16 | -6 | -2 |
|  | Thalamus | R |  | 17 |  | +2 | -10 | +6 |

^*^Significant at *p*_FWE_≤.05. ^**^Significant at *p*_FWE_≤.03. NEC=non-trauma-exposed controls. PTSD=posttraumatic stress disorder. BLA=basolateral subnucleus. SFA=superficial nucleus.

**Table S6.** Linear mixed effects modelling the effect of comorbidity of generalized anxiety (GAD) on amygdala functional connectivity for left hemisphere Cluster 1.

|  | | **Connectivity Score** | | |
| --- | --- | --- | --- | --- |
| *Predictors* | *Estimates* | | *CI* | *p* |
| (Intercept) | 0.00 | | -0.04 – 0.05 | 0.886 |
| ROI [CMA.L] | -0.05 | | -0.09 – -0.02 | **0.003** |
| ROI [SFA.L] | -0.02 | | -0.06 – 0.01 | 0.208 |
| GAD [Yes] | 0.01 | | -0.06 – 0.09 | 0.715 |
| **Random Effects** | | | | |
| σ^2^ | 0.01 | | | |
| τ_00_ _ID_ | 0.02 | | | |
| ICC | 0.62 | | | |
| N _ID_ | 65 | | | |
| Observations | 195 | | | |
| Marginal R^2^ / Conditional R^2^ | 0.019 / 0.626 | | | |

**Table S7.** Linear mixed effects modelling the effect of comorbidity of generalized anxiety (GAD) on amygdala functional connectivity for left hemisphere Cluster 2.

|  | **Connectivity Score** | | |
| --- | --- | --- | --- |
| *Predictors* | *Estimates* | *CI* | *p* |
| (Intercept) | 0.31 | 0.27 – 0.35 | **<0.001** |
| ROI [CMA.L] | -0.09 | -0.12 – -0.06 | **<0.001** |
| ROI [SFA.L] | 0.01 | -0.02 – 0.04 | 0.483 |
| GAD [Yes] | -0.02 | -0.08 – 0.05 | 0.584 |
| **Random Effects** | | | |
| σ^2^ | 0.01 | | |
| τ_00_ _ID_ | 0.01 | | |
| ICC | 0.58 | | |
| N _ID_ | 65 | | |
| Observations | 195 | | |
| Marginal R^2^ / Conditional R^2^ | 0.100 / 0.624 | | |

**Table S8.** Linear mixed effects modelling the effect of comorbidity of generalized anxiety (GAD) on amygdala functional connectivity for right hemisphere Cluster 1.

|  | **Connectivity Score** | | |
| --- | --- | --- | --- |
| *Predictors* | *Estimates* | *CI* | *p* |
| (Intercept) | 0.08 | 0.04 – 0.11 | **<0.001** |
| ROI [CMA.R] | -0.06 | -0.09 – -0.03 | **<0.001** |
| ROI [SFA.R] | -0.02 | -0.05 – 0.01 | 0.135 |
| GAD [Yes] | -0.05 | -0.10 – 0.01 | 0.134 |
| **Random Effects** | | | |
| σ^2^ | 0.01 | | |
| τ_00_ _ID_ | 0.01 | | |
| ICC | 0.56 | | |
| N _ID_ | 63 | | |
| Observations | 189 | | |
| Marginal R^2^ / Conditional R^2^ | 0.057 / 0.588 | | |

**Table S9.** Linear mixed effects modelling the effect of comorbidity of generalized anxiety (GAD) on amygdala functional connectivity for right hemisphere Cluster 2.

|  | **Connectivity Score** | | |
| --- | --- | --- | --- |
| *Predictors* | *Estimates* | *CI* | *p* |
| (Intercept) | 0.32 | 0.28 – 0.36 | **<0.001** |
| ROI [CMA.R] | 0.00 | -0.03 – 0.03 | 0.842 |
| ROI [SFA.R] | 0.07 | 0.04 – 0.10 | **<0.001** |
| GAD [Yes] | -0.05 | -0.11 – 0.02 | 0.159 |
| **Random Effects** | | | |
| σ^2^ | 0.01 | | |
| τ_00_ _ID_ | 0.01 | | |
| ICC | 0.61 | | |
| N _ID_ | 63 | | |
| Observations | 189 | | |
| Marginal R^2^ / Conditional R^2^ | 0.079 / 0.637 | | |

**Table S10.** Linear mixed effects modelling the effect of comorbidity of major depression (MDD) on amygdala functional connectivity for left hemisphere Cluster 1.

|  | **Connectivity Score** | | |
| --- | --- | --- | --- |
| *Predictors* | *Estimates* | *CI* | *p* |
| (Intercept) | 0.02 | -0.03 – 0.07 | 0.496 |
| ROI [CMA.L] | -0.05 | -0.09 – -0.02 | **0.003** |
| ROI [SFA.L] | -0.02 | -0.06 – 0.01 | 0.208 |
| MDD [Yes] | -0.02 | -0.09 – 0.05 | 0.529 |
| **Random Effects** | | | |
| σ^2^ | 0.01 | | |
| τ_00_ _ID_ | 0.02 | | |
| ICC | 0.62 | | |
| N _ID_ | 65 | | |
| Observations | 195 | | |
| Marginal R^2^ / Conditional R^2^ | 0.022 / 0.626 | | |

**Table S11.** Linear mixed effects modelling the effect of comorbidity of major depression (MDD) on amygdala functional connectivity for left hemisphere Cluster 2.

|  | **Connectivity Score** | | |
| --- | --- | --- | --- |
| *Predictors* | *Estimates* | *CI* | *p* |
| (Intercept) | 0.29 | 0.25 – 0.34 | **<0.001** |
| ROI [CMA.L] | -0.09 | -0.12 – -0.06 | **<0.001** |
| ROI [SFA.L] | 0.01 | -0.02 – 0.04 | 0.483 |
| MDD [Yes] | 0.02 | -0.04 – 0.08 | 0.445 |
| **Random Effects** | | | |
| σ^2^ | 0.01 | | |
| τ_00_ _ID_ | 0.01 | | |
| ICC | 0.58 | | |
| N _ID_ | 65 | | |
| Observations | 195 | | |
| Marginal R^2^ / Conditional R^2^ | 0.103 / 0.624 | | |

**Table S12.** Linear mixed effects modelling the effect of comorbidity of major depression (MDD) on amygdala functional connectivity for right hemisphere Cluster 1.

|  | **Connectivity Score** | | |
| --- | --- | --- | --- |
| *Predictors* | *Estimates* | *CI* | *p* |
| (Intercept) | 0.03 | -0.01 – 0.07 | 0.104 |
| ROI [CMA.R] | -0.06 | -0.09 – -0.03 | **<0.001** |
| ROI [SFA.R] | -0.02 | -0.05 – 0.01 | 0.135 |
| MDD [Yes] | 0.06 | 0.01 – 0.11 | **0.018** |
| **Random Effects** | | | |
| σ^2^ | 0.01 | | |
| τ_00_ _ID_ | 0.01 | | |
| ICC | 0.55 | | |
| N _ID_ | 63 | | |
| Observations | 189 | | |
| Marginal R^2^ / Conditional R^2^ | 0.090 / 0.587 | | |

**Table S13.** Linear mixed effects modelling the effect of comorbidity of major depression (MDD) on amygdala functional connectivity for right hemisphere Cluster 2.

|  | **Connectivity Score** | | |
| --- | --- | --- | --- |
| *Predictors* | *Estimates* | *CI* | *p* |
| (Intercept) | 0.29 | 0.25 – 0.34 | **<0.001** |
| ROI [CMA.R] | 0.00 | -0.03 – 0.03 | 0.842 |
| ROI [SFA.R] | 0.07 | 0.04 – 0.10 | **<0.001** |
| MDD [Yes] | 0.03 | -0.03 – 0.09 | 0.280 |
| **Random Effects** | | | |
| σ^2^ | 0.01 | | |
| τ_00_ _ID_ | 0.01 | | |
| ICC | 0.61 | | |
| N _ID_ | 63 | | |
| Observations | 189 | | |
| Marginal R^2^ / Conditional R^2^ | 0.070 / 0.637 | | |

**Table S14.** Linear mixed effects modelling partial volume effects of amygdala subnuclei on functional connectivity for left hemisphere Cluster 1.

|  | **Connectivity Score** | | |
| --- | --- | --- | --- |
| *Predictors* | *Estimates* | *CI* | *p* |
| (Intercept) | 0.15 | 0.04 – 0.26 | **0.007** |
| Group [PTSD] | -0.12 | -0.17 – -0.06 | **<0.001** |
| Condition [CMA.L] | -0.09 | -0.21 – 0.02 | 0.116 |
| Condition [SFA.L] | -0.09 | -0.19 – 0.01 | 0.092 |
| Age | -0.00 | -0.00 – 0.00 | 0.260 |
| Volume | 0.01 | -0.03 – 0.06 | 0.557 |
| Group [PTSD] × Condition [CMA.L] | 0.07 | 0.02 – 0.12 | **0.005** |
| Group [PTSD] × Condition [SFA.L] | 0.09 | 0.04 – 0.14 | **<0.001** |
| **Random Effects** | | | |
| σ^2^ | 0.01 | | |
| τ_00_ _ID_ | 0.01 | | |
| ICC | 0.59 | | |
| N _ID_ | 129 | | |
| Observations | 387 | | |
| Marginal R^2^ / Conditional R^2^ | 0.113 / 0.635 | | |

**Table S15.** Linear mixed effects modelling partial volume effects of amygdala subnuclei on functional connectivity for left hemisphere Cluster 2.

|  | **Connectivity Score** | | |
| --- | --- | --- | --- |
| *Predictors* | *Estimates* | *CI* | *p* |
| (Intercept) | 0.27 | 0.17 – 0.37 | **<0.001** |
| Group [PTSD] | 0.08 | 0.03 – 0.13 | **0.003** |
| Condition [CMA.L] | -0.01 | -0.12 – 0.10 | 0.846 |
| Condition [SFA.L] | 0.09 | 0.00 – 0.19 | **0.047** |
| Age | -0.00 | -0.00 – 0.00 | 0.459 |
| Volume | -0.01 | -0.06 – 0.03 | 0.583 |
| Group [PTSD] × Condition [CMA.L] | -0.11 | -0.15 – -0.06 | **<0.001** |
| Group [PTSD] × Condition [SFA.L] | -0.11 | -0.15 – -0.06 | **<0.001** |
| **Random Effects** | | | |
| σ^2^ | 0.01 | | |
| τ_00_ _ID_ | 0.01 | | |
| ICC | 0.61 | | |
| N _ID_ | 129 | | |
| Observations | 387 | | |
| Marginal R^2^ / Conditional R^2^ | 0.103 / 0.654 | | |

**Table S16.** Linear mixed effects modelling partial volume effects of amygdala subnuclei on functional connectivity for right hemisphere Cluster 1.

|  | **Connectivity Score** | | |
| --- | --- | --- | --- |
| *Predictors* | *Estimates* | *CI* | *p* |
| (Intercept) | -0.06 | -0.15 – 0.02 | 0.159 |
| Group [PTSD] | 0.07 | 0.03 – 0.12 | **0.001** |
| Condition [CMA.R] | 0.09 | -0.00 – 0.18 | 0.054 |
| Condition [SFA.R] | 0.14 | 0.06 – 0.22 | **0.001** |
| Age | 0.00 | -0.00 – 0.00 | 0.894 |
| Volume | 0.04 | -0.00 – 0.08 | 0.072 |
| Group [PTSD] × Condition [CMA.R] | -0.07 | -0.11 – -0.03 | **<0.001** |
| Group [PTSD] × Condition [SFA.R] | -0.09 | -0.13 – -0.05 | **<0.001** |
| **Random Effects** | | | |
| σ^2^ | 0.01 | | |
| τ_00_ _ID_ | 0.01 | | |
| ICC | 0.61 | | |
| N _ID_ | 126 | | |
| Observations | 378 | | |
| Marginal R^2^ / Conditional R^2^ | 0.057 / 0.636 | | |

**Table S17.** Linear mixed effects modelling partial volume effects of amygdala subnuclei on functional connectivity for right hemisphere Cluster 2.

|  | **Connectivity Score** | | |
| --- | --- | --- | --- |
| *Predictors* | *Estimates* | *CI* | *p* |
| (Intercept) | 0.29 | 0.19 – 0.38 | **<0.001** |
| Group [PTSD] | 0.09 | 0.04 – 0.14 | **<0.001** |
| Condition [CMA.R] | 0.08 | -0.02 – 0.18 | 0.122 |
| Condition [SFA.R] | 0.13 | 0.04 – 0.22 | **0.005** |
| Age | -0.00 | -0.00 – 0.00 | 0.298 |
| Volume | -0.03 | -0.07 – 0.02 | 0.235 |
| Group [PTSD] × Condition [CMA.R] | -0.14 | -0.18 – -0.09 | **<0.001** |
| Group [PTSD] × Condition [SFA.R] | -0.11 | -0.15 – -0.06 | **<0.001** |
| **Random Effects** | | | |
| σ^2^ | 0.01 | | |
| τ_00_ _ID_ | 0.01 | | |
| ICC | 0.58 | | |
| N _ID_ | 126 | | |
| Observations | 378 | | |
| Marginal R^2^ / Conditional R^2^ | 0.171 / 0.651 | | |
